# Ramadan and the Iftar Meal: A Qualitative Exploration of Signs of Disordered Eating in Muslim Men and Women Living in the United Kingdom

**DOI:** 10.1002/jclp.70119

**Published:** 2026-02-24

**Authors:** Alina Zubair, Lucy Hale, Sarfraz Jeraj, Lydia Poole

**Affiliations:** ^1^ Department of Psychological Interventions, School of Psychology University of Surrey Guildford UK; ^2^ Department of Psychology, Institute of Psychiatry, Psychology and Neuroscience King′s College London London UK

**Keywords:** disordered eating, eating disorders, Iftar, Muslim, qualitative, Ramadan

## Abstract

**Objective:**

To qualitatively explore the lived experiences of fasting during Ramadan and the Iftar meal in Muslim adults with low self‐regulation (i.e., low ability to control) eating behavior.

**Methods:**

Semistructured interviews were conducted with Muslim young adults, scoring below the threshold for low/moderate self‐regulation on the Self‐Regulation of Eating Behaviour Questionnaire, (SREBQ) (Kliemann et al. 2016). Interviews took place post‐Ramadan 2023. Nineteen participants were interviewed (11 females, mean age 24.8 years). Alongside interview questions, vignettes were used to explore participants' experiences of disordered eating. Interviews were audio‐recorded and transcribed verbatim.

**Results:**

Three themes were developed through reflexive thematic analysis highlighting how experiences of Ramadan and the Iftar meal may lead to the emergence of signs of disordered eating: (1) “refocussing on food provision,” which describes the centrality of food and food consumption during Ramadan; (2) “the pressure to consume,” which focuses on heightened social expectations that led to overeating during the Iftar meal; (3) “psychological distress and managing unpleasant physical sensations,” which refers to the emotional response following overeating and how this could lead to purging.

**Conclusion:**

This study highlights how the Iftar meal represents a challenging time for adults with low‐self regulation of eating and for some is associated with signs of disordered eating. While findings cannot be generalized to the broader Muslim population due to the small sample size, this study suggests a need for further community research regarding the experiences of Muslims vulnerable to disordered eating behavior and how to support them during Ramadan.

## 1 Introduction

Clinical eating disorders are associated with impairments in mental and physical health, including anxiety and malnutrition (Himmerich et al. 2021). The typical age of onset for eating disorders is 17–22 years (Solmi et al. 2022) and the aetiology includes a transition from disordered eating to eating disorder onset (National Eating Disorders Collaboration 2015; Puhl et al. 2014). Disordered eating includes eating behaviors, such as purging, but these are considered less severe or frequent than behaviors observed in eating disorders and are below the threshold for an eating disorder diagnosis (National Eating Disorders Collaboration 2015). Intermittent fasting, a key disordered eating behavior, involves abstaining from food for a prescribed period and then eating within the remaining hours (Stice et al. 2017). Intermittent fasting has been associated with the development of eating disorders, particularly bulimia nervosa (Stice et al. 2008, 2017), but research investigating fasting as a form of dietary restraint in relation to disordered eating/eating disorders is lacking.

Dietary restraint involves the ability to self‐regulate around food; during periods of fasting, self‐regulation is the process through which the impulse to consume food and drink is suppressed (Johnson et al. 2012). This is particularly salient within the context of eating disorders, where lower self‐regulation can lead to patterns of bingeing and/or bingeing and purging (Polivy and Herman 2002). In contrast, higher self‐regulation (e.g., excessive restriction) around eating can be a feature of eating disorders such as anorexia nervosa (Fairburn and Harrison 2003).

Individual motivations for intermittent fasting can vary from managing stress to religious observance. Religiosity (an individual′s dedication to religious belief [Mattis and Jagers 2001]) is underexplored in the disordered eating literature. Some studies report that higher religiosity is associated with an elevated risk of developing eating disorders (Richards et al. 2013; Thomas et al. 2018), while others have found the opposite (Doumit et al. 2017; Latzer et al. 2007; Sipilä et al. 2017). Most research has focused on Judaism or Christianity (Doumit et al. 2017; Latzer et al. 2007; Richards et al. 2013; Sipilä et al. 2017), with few studies focusing on disordered eating in Islam (Akgül et al. 2014; Düzçeker et al. 2021; Erol et al. 2008). Since Islamic practices include intermittent fasting, further research is needed to explore the role of observance of Islam in disordered eating/eating disorders.

Ramadan, the ninth month of the Holy Islamic Lunar Calendar lasts for 29–30 days and is one of Islam's five pillars (The Quran; 2:185). Observed worldwide, it involves abstaining from drinking and eating from sunrise to sunset (Rashed 1992), with the postfast meal called Iftar. During Ramadan, individuals with lower levels of self‐regulation around eating may be particularly challenged by extended periods of fasting. These individuals may, therefore, be at increased risk of disordered eating behaviors, particularly bingeing or bulimic type presentations; however, research in this area is scarce.

Indeed, few studies have investigated the impact of Ramadan on eating behaviors more generally. Some studies have found no harmful effects (Chia et al. 2018; Düzçeker et al. 2021), whereas others have found that religious fasting can trigger eating disorders (Akgül et al. 2014). However, most research has focused on Muslim‐majority countries, with few studies (e.g., Chia et al. 2018) in Western countries where Muslims are a minority. This is significant because cultural expectations and Western acculturation are likely to influence the prevalence of eating disorders (Song et al. 2023). Moreover, prior studies have neglected the male experience of eating disorders, and this is particularly salient since eating disorders are typically underdiagnosed, undertreated, and misunderstood in men (Strother et al. 2012). Finally, existing research has overlooked the specific experience of Iftar, which may provide important insights into the impact of fasting on subsequent (disordered) eating behavior.

The present study aims to qualitatively explore the experiences of fasting and eating during Ramadan in young UK Muslim men and women with reduced self‐regulation of eating behavior, with a particular focus on the emergence of signs of disordered eating. The objectives of the study were to:

1. Explore how UK Muslims with low eating behavior self‐regulation experience the transition from fasting to eating at Iftar during Ramadan.
2. Explore how Muslims manage changes to their eating behavior during Ramadan and Iftar.
3. Understand the emergence of disordered eating experiences during Ramadan.

## 2 Methods

### 2.1 Participant Selection and Recruitment

Participants were recruited opportunistically via social media platforms and through University Islamic Societies across the United Kingdom. Recruitment took place in the months prior to Ramadan (i.e., December 2022 to April 2023). Interested participants completed an eligibility screening questionnaire prior to the interview. This questionnaire screened against the inclusion criteria and if eligible, participants completed the SREBQ (Kliemann et al. 2016). The SREBQ has been found to consistently and reliably measure eating self‐regulation capacity (Kliemann et al. 2016). It is a short, 8‐item measure which has been found to show high internal consistency in previous studies (Cronbach′s *α* = 0.75; Kliemann et al. 2016). Items were summed, with items B and D reverse‐scored, to generate a total score ranging from 5 to 25. From the total score, the mean score was calculated and following Kliemann et al. (2016) scores ranging from < 2.8, 2.8–3.6, and > 3.6 were deemed to reflect “low,” “medium” or “high” levels of self‐regulation, respectively. Only those individuals with “low” or “medium” self‐regulation (i.e., scores ≤3.6) were eligible for the study, as it is thought that those with low self‐regulation are more likely to exhibit signs of disordered eating, for example binge eating (Ivezaj et al. 2016). Additional eligibility criteria included: identifying as Muslim; living in the United Kingdom; planning to fast during Ramadan 2023; aged 18–35 years. This age range was selected given the typical age of onset for eating disorders is 18–25 years (Allen et al. 2013; Volpe et al. 2016), with new eating disorders rarely presenting after 30 years (Micali et al. 2013) and illness duration lasting up to 6 years (Austin et al. 2021).

Malterud et al. (2016) suggest that “information power” should be used to determine sampling adequacy. Information power looks at the study aim, sample specificity, use of established theory, quality of dialog and analysis strategy to determine sample size. After 19 interviews, the research team were satisfied that the study met these criteria and a sufficiently rich data set had been generated.

### 2.2 Data Collection

This study used an exploratory qualitative design. Following the collection of informed consent, participants were asked to provide demographic information (e.g., age, gender, and ethnicity). Participants were not required to disclose their eating disorder history. Subsequently, one‐to‐one semistructured interviews were conducted. All interviews were conducted following the end of Ramadan 2023 over a 7‐week period. The interviews lasted for 51 min on average (range 34–80 min). All interviews were conducted online by the first author (A. Z.), a female trainee clinical psychologist with training in qualitative methods. A. Z. had no prior connection with participants, other than the contact made during the recruitment process where the study objectives and procedures were explained. The interview schedule was developed by the research team and was reviewed by the Service User and Carers Group at the University to ensure questions were asked clearly and sensitively. The interview focused on participants' general Ramadan experience and their specific experience around the Iftar meal. Participants were also shown three vignettes during the interview. These vignettes, written in the third person, contained three fictional accounts (one character per vignette) displaying signs of binge eating, bingeing and purging, and restrictive eating at Iftar, respectively. Vignettes were shown half‐way through the interview schedule and were included as an established method to elicit details from participants on sensitive topics (Tremblay et al. 2022). A copy of the interview schedule is provided in the Supporting Information. The interviewer kept a reflective diary to document thoughts, feelings and other interesting contextual information that occurred to her. Participants were provided with relevant local and national mental health service information and a £10 retail voucher honorarium. Interviews were audio‐recorded, with brief field notes taken, and transcribed verbatim by A. Z. No participants requested to check their transcripts. The project received ethical approval from a UK University Ethics Committee (FHMS 22‐23 005 EGA).

### 2.3 Data analysis

Reflexive thematic analysis (TA) was used to analyse the transcripts (Braun and Clarke 2019). Reflexive TA allows for a flexible approach, suggesting that themes are generated through participant and researcher interactions. This stance aligns with a social constructionist lens as it views knowledge as being co‐created through social and cultural interactions. The positionalities of the researchers were acknowledged as having an influence on the co‐construction of themes and the interaction between their experiences and the participants' narratives.

The data arising from the discussion of vignettes was not separated from the rest of the interview data, but rather the entire transcript was treated as a single data piece for analysis. The first author (A. Z.) read the transcripts and listened to the audio recordings to inductively generate codes and themes. A subsample of the transcripts were also read by L. H. and L. P. A. Z. reviewed each interview recording twice to ensure absorption and engagement with the data. A. Z. generated the codes and through discussion with L. H. (a clinical psychologist with a specialist interest in eating disorders) and L. P. (a health psychologist) and these were adapted and refined to incorporate multiple perspectives, contributing to a richer analysis. The coding tree included identification of specific emotional cues in response to fasting, relational aspects of Iftar and meal preparation, religious scripture as well as signs and symptoms of disordered eating. Coding of transcripts was performed using NVivo 12 (Lumivero, version 14). After the codes had been developed, an iterative approach was taken to organize the codes into themes and were refined through discussion and piloting. A. Z.'s reflective diary was consulted to ensure no additional meaning or interpretation could be gleaned from the data. Participants were randomly allocated pseudonyms to ensure anonymity. Data are presented as direct participant quotes, indicated in italics, with their pseudonym and scoring on the SREBQ denoted in parentheses.

## 3 Findings

In total, 71 individuals were screened for inclusion in the study, 47 were excluded as they did not meet eligibility criteria, and a further 5 participants did not respond to the invitation for an interview. Nineteen participants were eligible and took part in the interviews. Of these, 11 (58%) identified as women. Five (26%) participants were University students, while the remainder were employed in professional roles including medicine, optometry and law. Regarding ethnicity, 10 (53%) identified as Asian/Asian British–Pakistani and 11 (58%) participants were born in the United Kingdom (see Table 1 for an overview of participants' demographics).

**Table 1:** Demographic data, ranked by SREBQ score.

Pseudonym	Age (Years)	Gender	Ethnicity	SREBQ score	Classification on SREBQ	No. in household (incl. participant)
Eiman	26	Female	Asian/Asian British–Indian	2.2	Low	5
Yousuf	26	Male	Asian/Asian British–Pakistani	2.2	Low	4
Tayab	26	Male	Asian/Asian British–Pakistani	2.2	Low	3
Bilal	22	Male	Asian/Asian British–Pakistani	2.2	Low	6
Sara	21	Female	Asian/Asian British–Pakistani	2.4	Low	6
Rania	21	Female	Black/African/Caribbean/Black British–African	2.4	Low	2
Waleed	27	Male	Asian/Asian British–Pakistani	2.6	Low	5
Daniyal	21	Male	Asian/Asian British–Indian	2.8	Medium	3
Omar	29	Male	Asian/Asian British–Pakistani	3	Medium	1
Mahnoor	24	Female	Asian/Asian British–Pakistani	3	Medium	4
Inaya	20	Female	Black/African/Caribbean/Black British–African	3	Medium	5
Qasim	31	Male	Prefer not to say	3.2	Medium	1
Hiba	22	Female	Any other mixed/multiple ethnic background	3.2	Medium	4
Khadija	29	Female	Asian/Asian British–Pakistani	3.2	Medium	3
Laiba	25	Female	Asian/Asian British–Pakistani	3.2	Medium	5
Naila	21	Female	Any other Asian background	3.4	Medium	4
Zishaan	25	Male	Any other Asian background	3.6	Medium	5
Fatima	29	Female	Any other ethnic group	3.6	Medium	2
Ayesha	24	Female	Asian/Asian British–Pakistani	3.6	Medium	4

Abbreviation: SREBQ = Self‐Regulation of Eating Behaviour Questionnaire.

### 3.1 Thematic Overview

**Figure 1:**
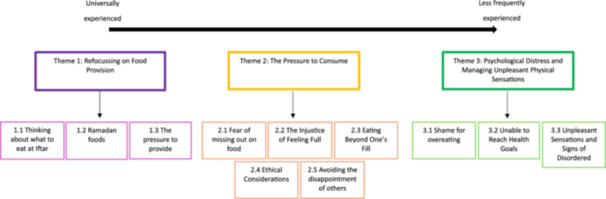
Thematic map.

The analysis produced three overarching themes as to how experiences of Ramadan and the Iftar meal might have led to the emergence of signs of disordered eating in individuals scoring in the low to medium range on the SREBQ. The first theme addressed our first objective to understand experiences of the transition from fasting to eating and highlighted the sociocultural mechanisms that led participants to prepare too much food for Iftar. The second theme was linked to our second objective to explore changes to eating behavior, discussing the internal and external pressures that acted as drivers for overeating. The final theme related to our third objective to understand the emergence of disordered eating and described the physical and psychological consequences of overeating and how some participants managed these feelings (see Figure 1 for a thematic map).

## 4 **Theme 1: Refocussing on Food Provision** “In Ramadan Obviously Eating Is a Big Deal” (Omar, Medium)

This theme reflected the paradox of Ramadan where Iftar represented a shift from fasting to eating. It described the centrality of food through the process of meal planning, discussions about food with family and friends, and food preparation. It spoke to the specialness of Ramadan and the additional effort put into providing food and meals to be enjoyed with others; in this way participants conveyed an excitement around eating.

1.1 *Thinking about what to eat at Iftar—*“Normally kind of we just set a menu at the start of the week…” (Yousuf, low).

Although Iftar occurs in the evening and food was only consumed at this time to break the fast, the meal planning, food shopping and food preparation started much earlier than this. Meals were considered, thought‐out and seen to be the focus of whole family discussions. This lasted throughout the fasting day, reflecting a sustained consideration of food. Participants described that “for Iftar, we make something different every other day or something… we might like, try new dishes… and er put a lot more effort into what we′re making” (Sara, low). Preparation of food was a planned activity because “as a family we start to prepare things half an hour to an hour before” (Naila, medium). By preparing food in advance of sunset to ensure that it is ready to be consumed, and making sure that dishes are new and not repeated, participants conveyed the significance of eating during Ramadan.

1.2. *Ramadan Foods—*“you don′t usually have it [food] like normally all year round” (Daniyal, medium)

Most participants (*n* = 10) described that food preparation was beyond their immediate control, either being prepared by mothers/sisters/wives, or for those who lived independently, being provided by Islamic societies. Compared to their lives outside of Ramadan, participants generally felt that there was a larger quantity of food available and greater effort was put into making dishes.

…So, um when I hosted Iftar, like all my friends brought a dessert dish … In Ramadan you make an effort to have these dishes available…

(Hiba, medium)

The extra effort to make dishes not typically eaten, made “Ramadan foods” special. Participants spoke about how eating for pleasure took precedence at Iftar, which was reflected in accounts of preparing samosas, pakoras, and other fried foods, typical of meals involving celebrations.

1.3 *The pressure to provide*—“And you will create an excess because you don′t want there to be a shortage…” (Khadija, medium).

Given the salience of food at Iftar, participants reported a pressure to provide large quantities of food; this was particularly apparent for Iftar parties.

…when you have an Iftar party, you've got so many people, you′re in this constant fear of “I don′t want there to be too little food”… So, you will try to cater for everyone that you can think of.

(Khadija, medium)

The need to ensure all guests can enjoy the Iftar meal highlighted the celebratory aspect of Iftar, which went beyond seeing food purely as sustenance. When holding Islamic practices in mind, as narrated by Jabbir bin Abdullah, the Prophet Muhammad (PBUH) said “the food of one person is sufficient for two, the food of two is sufficient for four, and the food for four is sufficient for eight” (Hadith 3254) (Ibn Majah nd‐c). This hadith suggests that whatever food is available can be shared. Muslims strive to follow the teachings of Prophet Muhammed (PBUH) and what is ordained in The Quran. Therefore, participants' concern to produce food at Iftar is inconsistent with Islamic scripture and teachings. However, the Prophet Muhammed (PBUH) was also described as being generous, particularly in Ramadan (narrated by Ibn Abbas; Hadith 3554) (Ibn Majah nd‐a). Our participants may have perceived generosity as “cater[ing] for everyone” (Khadija, medium) rather than sharing food with others. This shift in interpretation may be rooted in culture; some non‐Western cultures link hospitality with providing an abundance of food for guests (Sobh et al. 2013).

## 5 **Theme 2: The Pressure to Consume—**“I'm Not Really Feeling That [Food] but I'm Just Going to Have It Anyway, Just So They [Hosts] Know I Am Eating It and I Am Trying It Because I Don't Want to Upset Them” (Tayab, Low)

This theme referred to the internal and external sources of “pressure” that led individuals to overconsume food at Iftar. Internal sources of pressure included participants feeling fearful of missing out on foods, a lack of self‐control, and feeling a sense of injustice at being full quickly. The external sources of pressure stemmed from not wanting to waste food and not wanting to disappoint others by not eating.

2.1. *Fear of missing out on food—*“you see lots of different foods you get excited, and you want to sort of try it all.” (Daniyal, medium).

Overall, participants described “feel[ing] a fear of missing out” (Eiman, low) as they would not have the opportunity to eat Iftar dishes again until next Ramadan. This feeling was accompanied for some by a feeling of competing to obtain food.

…you know that you better have it now cuz otherwise you won′t get to have it erm so this is your chance to eat that … so you find room to have it… sometimes I will put everything on my plate… if there′s a lot of people around and if you don′t claim it, then it′s gonna go.

(Eiman, low)

As explained by Eiman, there is an element of having to “claim” Iftar foods so as not to miss out on eating foods perceived as “special.” This contributed to participants overfilling their plates and overeating: “…I′m kind of not really listening to my body so I′ll end up eating more…there are so many options so I want to try everything…” (Fatima, medium).

2.2. *The injustice of feeling full quickly—*“…you′ve fasted throughout the day and you feel that you deserve that food and you eat a lot and a lot and feel really full…” (Daniyal, medium)

Following the initial process of preparing food, serving food, and starting to eat, came the emotional response to eating. Many participants described an excitement to eat but felt full disappointingly quickly.

… those last few hours before Iftar, you′re just thinking about food and then that, yeah it′s almost disappointing actually that you get full so quickly because you′re thinking about it all day… I think the only motivation [to eat] is I′ve thought so long and hard about this during the day…

(Tayab, low)

Tayab described his anticipation to eat food before Iftar, only to be disappointed by not being able to consume as much as he imagined. This disappointment around feeling full implies a sense of entitlement to overeating at Iftar. In this way food is perceived as a reward for upholding the day's fast. This notion contradicts a hadith of the Prophet Muhammad (PBUH) narrated by Miqdam bin Madikarib which states that “a man does not fill any vessel worse than his stomach. It is sufficient for the son of Adam to eat what is enough for his body. But if he must do more than that, then one‐third for his food, one‐third for his drink and one‐third for his air” (Hadith 3349) (Ibn Majah nd‐d). According to religious teachings, while food is considered sacred it should not be the most prominent focus; overeating is not seen positively.

2.3. *Eating beyond one′s fill—*“when I am ready to break my fast I can eat as much as I possibly can… I eat a lot” (Waleed, low)

Factors such as the energy boost experienced after breaking their fast and concerns over needing to eat enough to feel sustained throughout the following day also contributed to descriptions of overeating by our participants. Moreover, many of our participants described a lack of self‐control at Iftar.

…if it was in front of me I would just eat it rather than thinking about what it was or if I was even like really wanting it like that cuz afterwards I′d be like I wasn′t even really craving it so why did I eat that much of it

(Inaya, medium)

This lack of self‐control around eating was interpreted to operate alongside our previously described themes of the increased availability of food and an increased sense of food entitlement to contribute to the overconsumption of food at Iftar. Inaya indicated that she ate automatically, without mindful presence. The lack of awareness when eating may have led participants to ignore or override physical satiety cues.

2.4. *Ethical considerations—*“…I′m quite big on not wasting food. So even if I don′t want to eat it, whatever I have ordered I will finish it or do my very best to finish it…” (Bilal, low)

Another factor that led to overeating was linked to moral obligation. Participants described not wanting to waste food, so felt compelled to eat it:

…I've got all the food on my plate and it's better if I stuff myself and clear my plate and finish it than end up wasting anything. But then at the same time, it's like I'm damned if I do, damned if I don't, because I feel bad either way… I waste the food, I feel terrible. I end up eating all it and I still feel terrible… There's no win.

(Rania, low)

Rania describes feeling conflicted, experiencing guilt regardless of the outcome. Across participants we observed that once someone committed to putting food on their plate, they felt tied to consuming this, as not finishing it would lead to intense feelings of guilt. This guilt was exacerbated if it was witnessed by other people.

But I feel like I would feel more shame if like I was around more people when this happened. But yeah. It′s probably more the guilt side

(Rania, low)

Participants spoke about feeling aware that there are many less fortunate people in the world who may not have access to food, so to waste it would be seen as shameful, as described by Laiba (medium): “…sometimes I feel like I don′t want to eat and I guess it′s that shame of oh I should eat not because I am hungry, but other people don′t have this, so I feel like that's where I relate to that in terms of the shame…”. In The Quran, it is heavily frowned upon to waste food: “O Children of Adam! wear your beautiful apparel at every time and place of prayer: eat and drink: But waste not by excess, for Allah loveth not the wasters” (The Quran, 7:31). Therefore, when considering the participants' behavior from an Islamic perspective, it seems that the fear of being wasteful perpetuated overeating.

2.5. *Avoiding the disappointment of others* “…I'll have a bit of everything to keep the hosts happy…” (Tayab, low)

Breaking the fast at Iftar led to conflicting emotions for participants. Some participants described a pressure to eat more food to not cause offence.

… Iftars where I would go to someone else's house, and you just can't say no to the food coz they're constantly supplying you with it… it′s guilt for not feeling rude and not eating what they have prepared for me. Especially if it's family or friends or those close friends' mum's who you consider to be family as well….

(Zishaan, medium)

As described by Zishaan, the need to eat was coupled with feelings of guilt arising from the recognition that the food had been prepared through the effort of another fasting individual. This suggests that consuming the Iftar food made by someone else is a sign of gratitude and politeness, both of which are valued Islamic traits and have been mentioned as a hadith of the Prophet Muhammed (PBUH) by Sinan bin Sannah al‐Aslami: “The one who eats gratefully has a reward similar to one who fasts patiently” (Hadith 1765) (Ibn Majah nd‐b).

## 6 **Theme 3: Psychological Distress and** Managing Unpleasant Physical Sensations “I′d Feel So Bloated My Stomach Would Be Really Like… Bloated Obviously That′s What It Is. And Um, Like, I Just… I Wouldn′t Feel Good Like I′d Just Feel Horrible…” (Inaya, Medium)

This theme recognized the psychological distress and physical discomfort which followed overeating at Iftar and how some participants managed these feelings. Feelings of shame and guilt were prevalent across the sample.

3.1. *Shame for Overeating—*“I felt like shameful like oh like I hate myself like oh you′re such an idiot, you ate that much… why did you do that.” (Tayab, low)

Many individuals felt shame for overeating, and this was observed as both internal and external sources of shame (Gilbert 1998). Internal shame refers to how one perceives oneself, whereas external shame reflects how one thinks others perceives oneself. Some participants spoke about the expectations and goals they had for themselves during Ramadan (e.g., in terms of maintaining/losing weight or gaining muscle) and by overeating they were unable to meet these goals. Being unable to uphold these expectations led to feelings of disappointment for some.

Because I would… have an expectation in my head or set myself a goal to do something or to not do something. So yeah, I′d be my own disappointment in myself basically.

(Mahnoor, medium)

Other feelings of shame stemmed from external sources such as weight stigma.

…there was that pressure that oh you should fit that specific size so, when they see you eating there′s you feel like your eyes… there are eyes on you if that kind of makes sense… Erm I do know there is a stigma about if you eat a lot if you′re a bit bigger, there is a lot of negativity around it in the communities.

(Sara, low)

… I wouldn't eat a lot at all because I know there are so many people. Like there, everyone is kind of around I'd feel as though people are watching me when I was eating, so I wouldn't eat anything. Like I would have a very small amount or avoid eating erm as much as I could.

(Inaya, medium)

Sara and Inaya described feeling self‐conscious of their body size, referring to an antifat bias among their social networks. While many participants were observed to overeat during Ramadan, some participants' experiences of shame and stigma were described alongside restrictive eating patterns. This contrast within our data highlights the complex interplay between social perceptions, body image and eating behaviors, whereby for some individuals the fear of judgment may override the otherwise common inclination to overeat at Iftar.

3.2. *Unable to reach physical health goals—*“…going to the gym is difficult while you′re fasting…” (Waleed, low)

Being unable to maintain or keep up with fitness/body image goals during Ramadan was another common experience that emerged across participants' accounts. Though this was not the case for all participants, male participants commonly reported concerns about preserving muscle mass and not losing too much weight.

…I would not lose too much muscle during that period…

(Zishaan, medium)

In contrast, female participants were more likely to have spoken about the importance of controlling their calorie intake at Iftar.

I'm not eating as much in my mind so you know technically it [weight] should go down… I was glad in a way because it didn't go up because that would′ve been a bit weird and a bit like oh why has it gone up.

(Laiba, medium)

One participant described that she would feel “pretty annoyed if I don′t stick to it [calorie limit], like it′s quite annoying and disappointing” (Mahnoor, medium). For some individuals, attempts to control calorie intake were accompanied by disappointment when this could not be achieved and this was described to have stemmed from “never lik[ing] the way my appearance and body is… I don′t′ feel great about it” (Inaya, medium). Overall, among female participants, the desire to avoid weight gain was a common concern, frequently underpinned by low body image.

3.3. *Unpleasant Sensations and Signs of Disordered Eating—*“…I′m kind of not really listening to my body so I′ll end up eating more…” (Fatima, medium)

Following the overconsumption of food during Iftar, some participants displayed signs of physical discomfort such as bloating and sickness.

…sometimes it felt like I'd overeaten, and I do feel a lot more bloated… I just don't feel good, like you know you feel a bit like your stomach is out here and you know it's like an underwhelming feeling. The anticipation just seems so much more exciting than that the actual sensation or feeling is when you get to that point.

(Tayab, low)

The feelings described by Tayab allude to physical discomfort, but also an emotional discomfort. This finding can be understood by previous research which has found that when individuals experience negative emotions, they are drawn to improve their emotional state (Lazarus 1991). The negative relief model suggests that an individual can alleviate negative moods through experiencing a positive reinforcing state (Cialdini et al. 1973). Therefore, in the context of experiencing shame and negative physical sensations (e.g., bloating), it follows that participants would attempt to alleviate such negative feelings. Eliminating food from the body may have felt like a reasonable step to tackle this negative feeling.

I ate too much… I've sort of purposely thrown up because I thought I already feel sick… then also in a way it's like the, the calories are kind of gone… and then it's with the intention of oh my god like I′ll be losing weight cuz as if I hadn't had dinner…

(Mahnoor, medium)

The only way that I will feel better is if this food comes out of me it's always usually just I… like… yeah, I go to the toilet straight away and I just try and pass it out… I would just, basically just sit on the toilet until something moves. I've done that before, I will literally just be sitting there even if I don't need to go, I′ll just be there until something goes basically.

(Rania, low)

Though these experiences were not shared by all participants, Mahnoor and Rania's experiences highlighted the extent to which some participants went to eradicate the effects of overeating by purging.

## 7 Discussion

This study aimed to understand British Muslims' experiences of Iftar during Ramadan and to explore the emergence of signs of disordered eating. Our findings highlighted three main themes, the first described how food is thought about at Iftar and how it is prepared, often resulting in an overproduction of food. The second theme reflected how participants experienced internal and external sources of pressure to consume the food that was made for Iftar, and how for many of the participants this led to overeating. The final theme highlighted how some participants experienced unpleasant physical sensations following overeating at Iftar and this led some participants to experience signs of disordered eating (e.g., purging) to cope. Across these themes those with both low and medium self‐regulation of eating behavior were represented, highlighting the broad commonality of experience across the sample.

The findings indicated that participants thought about their Iftar meals well ahead of time, in some cases several days in advance. Preoccupation with food is characteristic of disordered eating (Lydecker et al. 2022). It can be perceived as a way of obtaining control, and over prolonged periods, can lead to the development of eating disorders (Mathieu 2004). The food preoccupation observed in our participants does not necessarily indicate the development of an eating disorder; for example, thinking ahead about Iftar could be a practical way to ensure sufficient nutrition after fasting. However, for some, this early focus on food may signal a psychological preference for control, a trait linked to disordered eating (Froreich et al. 2016). Further research is needed to understand how meal planning and food preoccupation during Ramadan may present as an early warning sign for disordered eating.

Many participants discussed the specialness of Ramadan specific foods. This led some participants to overproduce and overconsume these foods at Iftar. Studies have found that repeated consumption of special foods can lead to senitization, meaning that the brain begins to anticipate rewards, leading to stronger cravings, which in turn can lead to overeating (Finlayson et al. 2007). In the case of Ramadan, these special foods hold emotional and cultural significance; as such, their novelty may enhance this sensitisation process. Prior research has also found that people typically eat more if they are feeling hungry (Vartanian et al. 2017). Additionally, to determine the appropriate level of food consumption, one relies on past experiences of eating and predictions of future satiety (Higgs 2005; Vinai et al. 2009). This suggests when satiety cannot be achieved, limited food availability can cause hunger to persist; the need to satisfy hunger via overproduction of food was widely shared by our participants.

Given the sample consisted of individuals with low‐medium self‐regulation of eating, our participants may have found it particularly difficult to regulate their intake of Ramadan foods. Duckworth et al. (2016) have proposed that self‐control weakens over time through a cycle of situational response. Within this context, individuals who were hungry and exhibited low‐medium self‐regulation may have been particularly susceptible to reduced self‐control and subsequent overeating at Iftar when presented with a surplus of food. The ICD‐11 (International Classification of Diseases‐11) criteria of binge eating stipulates the key symptom is a loss of control experienced when eating, alongside consumption of a large amount of food (defined as an objectively large amount of food in general, or a subjectively large amount of food for the individual) (Berner et al. 2020; Reed et al. 2019). The automaticity of eating was also experienced by our participants, and this has also been shown by some authors to lead to bingeing (Giannopoulou et al. 2020). Overall, the loss of control that some participants described in our study may be indicative of disordered eating; however, further research is needed to directly examine the extent to which these acute behaviors align with the clinical criteria for binge eating.

Another factor which contributed to participants' overeating was linked to concerns over food waste. Given that both excessive consumption and the wastage of food are proscribed within Islamic teachings, participants reported experiencing negative emotions in either circumstance. One mechanism that may drive feelings of failure is cognitive dissonance (Harmon‐Jones and Mills 2019). Cognitive dissonance may arise when one's beliefs or behaviors do not align with moral or religious teachings (Harmon‐Jones and Mills 2019). One approach to mitigate cognitive dissonance is the undertaking of deliberate sacrifices, a process that may, in turn, reinforce commitment (Aksoy and Gambetta 2022). In our study, participants described prioritizing a desire not to be wasteful, which in turn enabled them to justify overeating. Nevertheless, feelings of shame for overeating were common. Prior research has shown that external shame is more strongly related to psychopathology than internal shame, particularly depression and low mood (Kim et al. 2011), both of which are risk factors for developing eating disorders (Polivy and Herman 2002). Additionally, internal factors such as thin ideal internalization and shame susceptibility can contribute to signs of disordered eating (Nechita et al. 2021). It is not clear the extent to which these factors contributed to purging behavior in our study or indeed whether the instances of purging experienced were indicative of a wider pattern of disordered eating. However, our findings offer some preliminary insights into the importance of these processes for individuals during Ramadan.

### 7.1 Clinical implications

As an exploratory study, our results indicate that Ramadan and Iftar may present challenges for some Muslims living in the United Kingdom who have low‐to‐medium self‐regulation around eating. These preliminary findings suggest that individuals with lower self‐regulation may require additional support to navigate certain aspects of Iftar, such as abundance of food and communal eating; however, more work is needed to corroborate our findings. This study also highlights a potential need for increased mental health awareness regarding signs of disordered eating during Ramadan among Muslims (Ibrahim and Whitley 2021). For example, training healthcare providers to engage in culturally competent conversations about eating in Ramadan could represent an important first step in intervention development. Similarly, informing Islamic community leaders of these issues could support the longer‐term co‐production of culturally sensitive, evidence‐based resources. In light of our findings, one potential implication is to consider the adaption of existing evidence‐based treatments such as cognitive behavioral therapy (CBT) for individuals who experience signs of disordered eating during Ramadan. Behavior modification strategies to navigate food abundance could be one example. However, further research is needed to assess the acceptability, feasibility, and effectiveness of any such adaptations within this specific context.

### 7.2 Strengths and limitations

All participants were practicing Muslims and were interviewed shortly following Ramadan, reducing the risk of recall bias. The trustworthiness of our analysis, has been enhanced through researcher triangulation, drawing on the multiple expertise of the research team. Moreover, the analysis was conducted at multiple levels, beginning with reflective diaries and extending through the iterative process of theme generation. Participant checking was not undertaken which could have added a useful step to our interpretive work. Another strength is that this research uniquely explores Muslims in a minority context, which contrasts to prior studies which have focused on Muslims in majority settings. This provides valuable insight into how cultural environments may influence fasting and eating behaviors, particularly through the effects of minority pressures or acculturation. Additionally, previous research on fasting and religion has largely focused on Christian and Jewish populations. By exploring fasting in a Muslim context, this research broadens the literature, highlighting challenges and differences shaped by cultural and religious factors.

Nevertheless, some limitations must also be recognized. Our sample comprised data from 19, relatively young, participants, which means that it is not possible to generalize the findings to all Muslim men and women across the United Kingdom, or indeed globally. Moreover, the focus of this study was on those with low‐medium self‐regulation of eating. Lower levels of self‐regulation tend to be associated with some eating disorder subtypes, such as bulimia nervosa, while anorexia nervosa is generally characterized by higher levels of self‐regulation around eating, reflecting its focus on food restriction (Fairburn and Harrison 2003; Lavender et al. 2015; Polivy and Herman 2002). As such, experiences of more restrictive presentations of eating were not fully captured in our sample. It may be beneficial for future studies to examine individuals with high levels of self‐regulation and their relationship with eating behaviors during Ramadan.

## 8 Conclusion

This qualitative study explored how Muslims with low to medium self‐regulation around eating transition from fasting to eating during Ramadan, with a focus on experiences of disordered eating at Iftar. The findings indicated that signs of disordered eating can emerge during Iftar, often in response to increased food availability, leading to overeating and associated emotional and physical discomfort. These findings suggest further research is needed to explore the experiences of Muslims living with eating disorders and strategies to support them during Ramadan.

## Author Contributions

Alina Zubair formulated the research question alongside Lydia Poole. Alina Zubair collected the data, analyzed the data, interpreted the data, wrote and revised the manuscript. Lydia Poole interpreted the data and reviewed and revised the manuscript. Lucy Hale supported with data analysis and theoretical conceptualization. Lucy Hale and Sarfraz Jeraj reviewed and revised the manuscript.

## Funding

The authors received no specific funding for this work.

## Conflicts of Interest

The authors declare no conflicts of interest.

## Data Availability

Consent for data sharing was not obtained from participants so the data is not available for sharing.
